# Targeting Epidermal Growth Factor Receptor (EGFR) and Human Epidermal Growth Factor Receptor 2 (HER2) Expressing Bladder Cancer Using Combination Photoimmunotherapy (PIT)

**DOI:** 10.1038/s41598-019-38575-x

**Published:** 2019-02-14

**Authors:** Mohammad R. Siddiqui, Reema Railkar, Thomas Sanford, Daniel R. Crooks, Michael A. Eckhaus, Diana Haines, Peter L. Choyke, Hisataka Kobayashi, Piyush K. Agarwal

**Affiliations:** 1 Urologic Oncology Branch, Center for Cancer Research, National Cancer Institute, Bethesda, MD – 20892 USA; 2 Diagnostic and Research Services Branch, Office of the Director, National Institutes of Health, Bethesda, MD – 20892 USA; 3 Pathology Section, Pathology/Histotechnology Laboratory, Leidos Biomedical Research, Inc. Frederick National Laboratory for Cancer Research, Frederick, MD 21702 USA; 4 Molecular Imaging Program, Center for Cancer Research, National Cancer Institute, Bethesda, MD – 20892 USA; 5 Department of Urology, Northwestern University Feinberg School of Medicine, Chicago, IL USA

## Abstract

Bladder cancer (BC) is heterogeneous and expresses various cell surface targets. Photoimmunotherapy (PIT) involves monoclonal antibodies (MAbs) conjugated to a photoabsorber (PA), IR Dye 700Dx, and then activated by near infra-red light (NIR) to specifically target tumors. We have demonstrated that tumors expressing EGFR can be targeted with PIT. However, PIT may be less effective when a tumor lacks “overwhelming” expression of a single target such as EGFR. We present a combinatorial PIT approach for targeting BC expressing EGFR and HER2, using PA- labeled panitumumab (pan) and trastuzumab (tra), respectively. Human BC tissues and cell lines were analyzed for EGFR and HER2 expression. Efficacy of PA-labeled MAbs singly and in combination was analyzed. About 45% of BC tissues stain for both EGFR and HER2. *In vitro*, the combination of pan IR700 and tra IR700 with NIR was more efficacious than either agent alone. Tumor xenografts treated with combination PIT showed significant tumor growth retardation. Combination PIT is a promising approach for treating BC with low/moderate expression of surface receptors. In addition, given the molecular heterogeneity of bladder cancer, targeting more than one surface receptor may allow for more effective cell death across different bladder tumors.

## Introduction

Bladder cancer (BC) is the 4^th^ most common cancer in men and the 12^th^ most common cancer in women, and ranks 8^th^ in all cancer related deaths. It is estimated that in 2018, 81,190 new cases of BC will be diagnosed while accounting for 17,240 cancer-specific deaths^1^. Approximately 70% of cases are non-muscle invasive bladder cancer (NMIBC) at presentation and are treated by transurethral resection of bladder tumor (TURBT) followed by intravesical treatment with BCG (Bacillus Calmette-Guerin) or mitomycin C. However up to 70% of NMIBC recur after initial treatment, with 10–20% progressing to muscle-invasive bladder cancer (MIBC)^2^. MIBC is aggressive and only 50% of patients will survive five years despite undergoing radical cystectomy^3^. Unfortunately, no breakthrough treatments for localized bladder cancer have emerged in over two decades. The current therapeautic options have subpar efficacy, as reflected in high rates of recurrance, requring close BC surveillance from diagnosis to death. As a result, BC is one of the most expensive malignancies to treat^4^. Clearly, there is a large unmet need for better NMIBC therapeutic options.

Photoimmunotherapy (PIT) is a novel molecular-targeted cancer therapy that is based on a monoclonal antibody (MAb) conjugated to a photosensitizing phthalocyanine dye, IRDye700Dx (IR700), and its irradiation by near-infrared (NIR) light^5^. The MAb IR700 conjugate is directed against MAb-specific surface proteins enriched on the cancer cell membranes. Upon NIR delivery, activation of IR700 results in rapid rupture of the cell membrane where the MAb IR700 conjugate is bound while sparing the unbound normal cells^6^. Recent work has demonstrated that the effects of PIT, at any given dose of NIR light irradiation, can be enhanced by increasing the number of cell surface-bound molecules of MAb IR700^7^. Another approach that has demonstrated increased efficacy is combination PIT as shown when two different epitopes of human epidermal growth factor receptor 2 (HER2) were targeted on human gastric cancer cells using trastuzumab IR700 and pertuzumab IR700^8^.

The Cancer Genome Atlas (TCGA) project demonstrates that BC has the third highest mutation rate among all studied cancers, making it one of the most molecularly heterogeneous cancers^9^. It has been shown to have differential enrichment of cell surface proteins, including Epidermal Growth Factor Receptor (EGFR) and Human Epidermal Growth Factor Receptor – 2 (HER2), depending on the subtype^10^. EGFR overexpression is seen in up to 74% of bladder cancer tissue specimens, with a relatively low expression seen in normal urothelium^11,12^. HER2 overexpression is reported in up to 40% of bladder cancer specimens, with no expression found in normal urothelium^12^. Another series looking at EGFR and HER2 expression in human bladder cancer specimens found EGFR expression in up to 72.2% of tumors and HER2 expression in 44.5% of tumors, with 33.9% of tumors co-expressing both^13^.

Previously, we have reported that BC expressing high levels of cell surface EGFR can be effectively targeted using EGFR-directed PIT, both *in vitro* and *in vivo*^6^. However, such levels of cell surface EGFR expression are mainly seen in the basal subtype of BC, while the luminal subtype shows enrichment in cell surface HER2 expression among others^10^. Given that BC is molecularly heterogeneous with many tumors expressing only moderate levels of multiple cell surface targets, including EGFR and HER2, we hypothesize that such tumors can be selectively targeted using combination PIT directed against EGFR and HER2 concurrently. In this report, we employ an anti-EGFR MAb IR700 conjugate, panitumumab IR700 (pan IR700), and an anti-HER2 MAb IR700 conjugate, trastuzumab IR700 (tra IR700), to illustrate that combination PIT using EGFR and HER2 as co-targets is selective and effective in targeting such BC *in vitro* and *in vivo*.

## Methods

### Reagents

The two humanized monoclonal antibodies panitumumab (anti-EGFR) and trastuzumab (anti-HER2) were procured from Amgen (Thousand Oaks, CA) and Genentech (San Francisco, CA), respectively. IRDye 700Dx NHS ester (IR700) was purchased from LI-COR Biosciences (Lincoln, NE, USA). Alexa-488 and Alexa-594 antibody labeling kits were purchased from ThermoFisher Scientific, MA, USA. Phycoerythrin (PE) conjugated-Rat MAb against hEGFR as well as PE conjugated Rat IgG2a, kappa MAb (isotype control) were obtained from Abcam (Cambridge, MA). PE conjugated Mouse MAb IgG1 against hHER2 and mouse IgG1 conjugated to PE (isotype control) were purchased from Biolegend (San Diego, CA) and R&D systems (Minneapolis, MN), respectively. The CellTiter Glo Luminescent Cell Viability Assay was purchased from Promega (Madison, WI, USA). All other chemicals were reagent grade and purchased from Sigma Aldrich unless otherwise noted.

### Synthesis and purification of IR700-conjugated panitumumab and trastuzumab, Alexa488-conjugated panitumumab, Alexa594-conjugated trastuzumab

IR700 conjugation to panitumumab or trastuzumab was achieved by incubating the dye and antibody at a 3.1:1 ratio in 0.1 M Na_2_HPO_4_ (pH 8.5) at room temperature for 1 h. The mixture was purified using a Sephadex G50 (PD-10; GE Healthcare, Piscataway, NJ, USA). Panitumumab was conjugated to Alexa488 using the Alexa488 antibody labeling kit (Life Technologies Inc.). Similarly, trastuzumab conjugation to Alexa594 was achieved using the Alexa594 antibody labeling kit (Life Technologies Inc.). The number of fluorophore molecules per MAb molecule was adjusted to approximately 3 for IR700 and 4 for both Alexa488 and Alexa594.

### Cell Culture

The bladder cancer cell lines TCCSUP (HTB-5), 5637 (HTB-9), RT4 (HTB-2), T24 (HTB-4), ScaBER (HTB-3), HT1197 (CRL-1473), HT1376 (CRL-1472), SW780 (CRL-2169), NIH/3T3 (CRL-1658), and SK-BR-3 (HTB-30) were obtained from ATCC (Manassas, VA, USA). RT112 was obtained from DSMZ, Germany. Metastatic lines of T24 - T24T, FL3, and SLT3 were gifted by Dr. Michael Nickerson (Division of Cancer Epidemiology & Genetics, NCI). The bladder cancer cell lines 253 J, UMUC-5, and UMUC-1 were a kind gift from Dr. David McConkey (Johns Hopkins University). MGH-U3 was obtained from Massachusetts General Hospital. RT4v6 was a gift from Dr. Peter Black (University of British Columbia). All cells were grown in minimum essential media (MEM) (Life technologies) supplemented with 10% fetal bovine serum, 1% penicillin/streptomycin and 1% GlutaMAX (Life technologies). All cells were incubated in a humidified incubator at 37 °C with 5% CO_2_ for growth.

### Determination of HER2 and EGFR expression in bladder cancer

The Cancer Genome Atlas (TCGA) data were used to determine EGFR and HER2 expression based on BC subtypes. Data from TCGA were downloaded from its website (http://cancergenome.nih.gov/). Level 3 RNA seq count data were downloaded for all patients with bladder cancer and the data were transformed for linear modeling using the *voom* function in the limma R package^14^. 131 patients were included in the initial comprehensive characterization of urothelial carcinoma. 19 normal urothelial samples were also included in the TCGA bladder cancer provisional dataset to be used as a reference. Gene expression was evaluated within each of 4 subtypes as defined in the original TCGA publication – TCGA identifiers from the original publication were applied to the samples in the downloaded data to define the subtypes. Differences in mean expression between the 4 subtypes were evaluated with ANOVA.

EGFR and HER2 expression were also determined using tissue microarray (TMA). Two commercially available TMAs were purchased from Biomax (Rockville, MD): BL2081 and BL806. There were a total of 288 samples with 232 bladder cancer samples, 8 normal samples, and 48 samples of normal adjacent bladder cancer. Of the 232 samples that were bladder cancer, 86 (36%) were Ta/T1, 108 (47%) were T2, and 38 (16%) were T3. The majority of samples were pure urothelial histology (197, 85%), while 35 (15%) were variant histology - 7 adenocarcinoma, 10 mucinous adenocarcinoma, and 18 squamous tumors. Each sample was graded by a single pathologist (DH) for staining as follows: 0 = <10% of cells positive; 1 = 10–24%; 2 = 25–49%; 3 = 50–74%; 4 = 75–100%. The tissue microarrays were stained for the presence of EGFR using anti-hEGFR antibody (Cell Signaling Technology No. 4267). Separate tissue microarrays were stained for the presence of HER2 using the anti-HER2 antibody (Cell Signaling Technology No. 2165). For staining, slides were de-paraffinized and rehydrated with distilled water. Antigen retrieval was performed with 1 mM EDTA for 15 mins at 95 °C. Following a normal goat serum block, sections were incubated with the 1:100 diluted primary antibody overnight. Sections were rinsed and incubated with biotinylated goat anti-rabbit IgG, followed by ABC Elite reagent. DAB was used for detection. Slides were counterstained with hematoxylin and covered with coverslip. For the purposes of evaluating the EGFR and HER2 expression, staining was considered negative for grade 0 and positive for grade 1–4.

### Determination of EGFR and HER2 expression *in vitro* using flow cytometry

To determine EGFR and HER2 expression on the panel of human bladder cancer cell lines, PE fluorescence was measured using FACSCanto II flow cytometer (BD BioSciences) after incubation with PE conjugated anti-HER2 or anti-EGFR. Briefly, a single cell suspension of cells was prepared using trypsin and suspended in immunofluorescence assay (IFA) buffer at 1 × 10^6^ cells/100 uL of IFA buffer per reaction. PE-conjugated mouse IgG1 anti-HER2 (10 μL) or PE-conjugated rat IgG2a, κ anti-EGFR (10 μL) were added to each reaction and allowed to incubate for 1 h at room temperature. PE-conjugated mouse IgG1 (10 μL) and PE-conjugated rat IgG2a, κ (2 μL) were used as isotype controls, respectively. Cells were then washed with 1x phosphate buffered saline (PBS), and PE fluorescence was collected on flow cytometer. Data was analyzed using FlowJo (Treestar Inc.). The median fluorescence intensities (MFIs) were evaluated and compared to the median fluorescence intensity (MFI) of the isotype controls. The relative median fluorescence intensity (RMFI) for each cell line was calculated by the formula:

$$RMFI=\frac{Median\,Fluorescence\,Intensity\,(Anti-EGFR)\,or\,\,(Anti-Her2)}{Median\,Fluorescence\,Intensity\,(Corrosponding\,Isotype\,Control)}$$

A431 and NIH/3T3 cell lines were used as positive and negative controls, respectively, for EGFR expression. SK-Br-3 and NIH/3T3 cell lines were used as positive and negative controls, respectively, for HER2 expression.

### Determination of Panitumumab and Trastuzumab specificity and colocalization on SW780 cells using immunocytochemistry

Target specificity of panitumumab (EGFR) and trastuzumab (HER2) and their ability to co-localize on the same cells were confirmed using immunocytochemistry. Briefly, panitumumab was conjugated to Alexa-488 (Pan-Alexa488) and trastuzumab was conjugated to Alexa-594 (Tra-Alexa594). NIH-3T3 and SW780 cells were seeded at 3 × 10^4^ cells/well on a glass slide and incubated at 37 °C overnight. Cells were then fixed using 2% paraformaldehyde (PFA)/PBS at room temperature for 30 minutes, followed by a 30-minute incubation with 5% bovine serum albumin (BSA) in 1x PBS with 0.1% v/v Tween-20 (1x PBST) to block non-specific sites. Cells were then incubated for 3 hours with either 50 μg/mL panitumumab-Alexa488 (Pan-Alexa488) or 100 μg/mL of trastuzumab-Alexa594 (Tra-Alexa594) or both. Hoescht 33342 (1:1000) was used as a nuclear stain. All images were taken using (Keyence, BZ-X Analyzer, Fluorescence Microscope, Osaka, Japan) at the same camera setting.

### Determination of MAb-IR700 dose response using flow cytometry

To evaluate the IR700 fluorescence intensities of the cells after treatment with tra IR700 plus pan IR700 or either agent alone, flow cytometry analyses were performed. Single cell suspension of about 1 million cells/100 µl of IFA buffer per reaction were incubated with 0.1, 1, 10, 100 μg of tra IR700 or 0.1, 1, 10, 100 μg of pan IR700; or 0.1, 1, 10, 100 μg of both tra IR700 and pan IR700 at room temperature for 1 h. Cells were then washed with 1x PBS and MFIs were determined using the BD FACSCanto II flow cytometer.

### *In vitro* photoimmunotherapy (PIT)

Cells were seeded at 5 × 10^4^ cells/well in 35-mm dishes and incubated at 37° C for 24 h. The medium was then replaced by 1 mL of fresh, phenol-free media containing 15 μg (100 nM) of tra IR700, pan IR700, both tra IR700 and pan IR700, or 200 nM of IR700. Following 24 h incubation, cells were then irradiated with NIR light ranging from 0–100 J/cm^2^ (0, 4, 10, 20, 40, 64, 80, 100 J/cm^2^) using light-emitting diode, emitting light at 670–710 nm (L690-66-60; Epitex Inc., Kyoto, Japan). Cell viability was then determined 24 h later using the CellTiter Glo Luminescent Cell Viability Assay (Promega), and lethal dose 50 (LD_50_) for NIR was then determined using GraphPad Prism (GraphPad software, Inc.).

To confirm that cytotoxic effects were not from the antibody treatment itself, 5 × 10^3^ SW780 cells/well were seeded overnight in a 96-well plate. The media was then replaced with a fresh, phenol-free media containing no drug, 100 nM panitumumab, 100 nM trastuzumab, 100 nM panitumumab and 100 nM trastuzumab together, 100 nM pan IR700, 100 nM tra IR700, 100 nM pan IR700 and 100 nM tra IR700 together, or 200 nM IR700. Following 24 h and 72 h incubation, cell survival was assessed using the CellTiter Glo Luminescent Cell Viability Assay.

### *In vivo* photoimmunotherapy (PIT)

All animal studies were conducted in accordance with the Guide for Care and Use of Laboratory Animal Resources (1996), U.S. National Research Council, and approved by the local Animal Care and Use Committee, the NCI-Bethesda Animal Care and Use Committee (ACUC). Five-week old athymic Nu/nu female mice were purchased from Charles River Laboratory (Frederick National Laboratory, Frederick, MD). All animals were housed in a controlled light-dark cycle (12:12 h) room. About, 3 million SW780 cells re-suspended in 50% Matrigel (v/v) in 1X PBS were injected subcutaneously in the right thigh of the mice. The tumor xenografts were measured with an external caliper, and the tumor volume was calculated using the following formula: (length × width^2^) × 0.5. When the tumor volumes reached about 50–100 mm^3^, mice were randomized into 4 groups of 10 mice each, with groups balanced for mean and median tumor size. The treatment groups were as follows: Tumor only with NIR, pan IR700 with NIR, tra IR700 with NIR, and pan IR700 + tra IR700 with NIR. Each treatment regimen involved an antibody or PBS intravenous tail vein injection followed by initially 50 J/cm^2^ NIR irradiation 24 h later and then 100 J/cm^2^ NIR irradiation 24 h later in a separate experiment. In both experiments, on Day 0, mice received either 100 µL of PBS (tumor only with NIR) or 100 µg/100 µL of IR700-MAb with combination group receiving 100 µg + 100 µg of both antibodies/100 µL. This was followed by 50 J/cm^2^ NIR irradiation or 100 J/cm^2^ NIR irradiation on Day 1. The xenografts were measured with external calipers on Day 0 and twice a week after Day 0.

To study possible toxicity of the combination PIT therapy, a total of 14, 5-week-old athymic Nu/Nu mice were injected subcutaneously with 3 × 10^6^ SW780 cells (1:1 Matrigel: 1x PBS) in the right dorsal flank. Tumors were allowed to grow for a week until they reached 50–100 mm^3^. Out of 14 mice, 12 mice were then injected with 100 μg of pan IR700 + 100 μg of tra IR700, and 2 mice were injected with 100 μL of 1x PBS as controls. 100 J/cm^2^ NIR irradiation of tumors were then performed 24 h later. Two mice were then euthanized every 8 h over the course of 32 h. Heart, lungs, liver, kidneys, sternum (bone marrow sample), spleen, and tumor were collected and fixed using 10% buffered formalin. Pathology slides were prepared by HistoServ (Germantown, MD) and reviewed by a mouse pathologist, MAE (National Institutes of Health, Department of Veterinary Services, Bethesda, MD).

A similar experiment was performed to collect blood for chemical analysis including plasma electrolytes and other enzymes. Total of 10 Athymic Nu/Nu female mice growing SW780 tumors on their right flank were injected intravenously with either 1XPBS or with 100 μg of pan IR700 + 100 μg of tra IR700. Twenty-four hours later, the mice were irradiated with 100 J/cm^2^ of NIR. The mice were euthanized 8 hours later and blood was collected directly by puncturing the heart using 27 1/2 G needles into PST™ Microtainer^®^ tubes containing Lithium Heparin (BD, Franklin Lakes, NJ, USA). Blood was mixed properly, centrifuged at 5600RPM for 15 minutes to separate plasma. The plasma chemistry was performed using VetScan^®^ Comprehensive Diagnostic Profile (Abaxis, Inc., Union City, CA, USA).

### Lactate Dehydrogenase In-Gel Activity Assay

LDH isozymes in mouse plasma samples and tissues were separated by native gel electrophoresis using pre-cast 1 mm 6% Tris-Glycine polyacrylamide gels (Invitrogen). Briefly, 4 µL mouse plasma or 8 µg of tissue lysate were mixed with 5 µL of a solution consisting of 20% glycerol, 50 mm Tris, pH 8, and 0.2% bromophenol blue, 8 µL was loaded into the gel, and the samples were subjected to electrophoresis at room temperature for 1.25 hours at 125 V, with running buffer consisting of 25 mM Tris, 192 mM glycine, pH 8.3. Subsequently, the gels were immersed in 10 mL of a solution pre-warmed to 37 °C, consisting of 50 mM Tris, pH 7.4, 1 mg/mL iodonitrotetrazolium bromide (INT), 1 mM NAD+, 0.184 mg/mL phenazine methosulfate (PMS), and 5mM L-lactate all dissolved in deionized water. The gels were gently rocked at 37 °C in the dark for approximately 5 minutes, until LDH enzyme activity bands were developed, at which point the gels were de-stained in the dark with sequential incubations in de-ionized water until the background was clear.

## Results

### EGFR and HER2 expression in human bladder cancers

**Figure 1:**
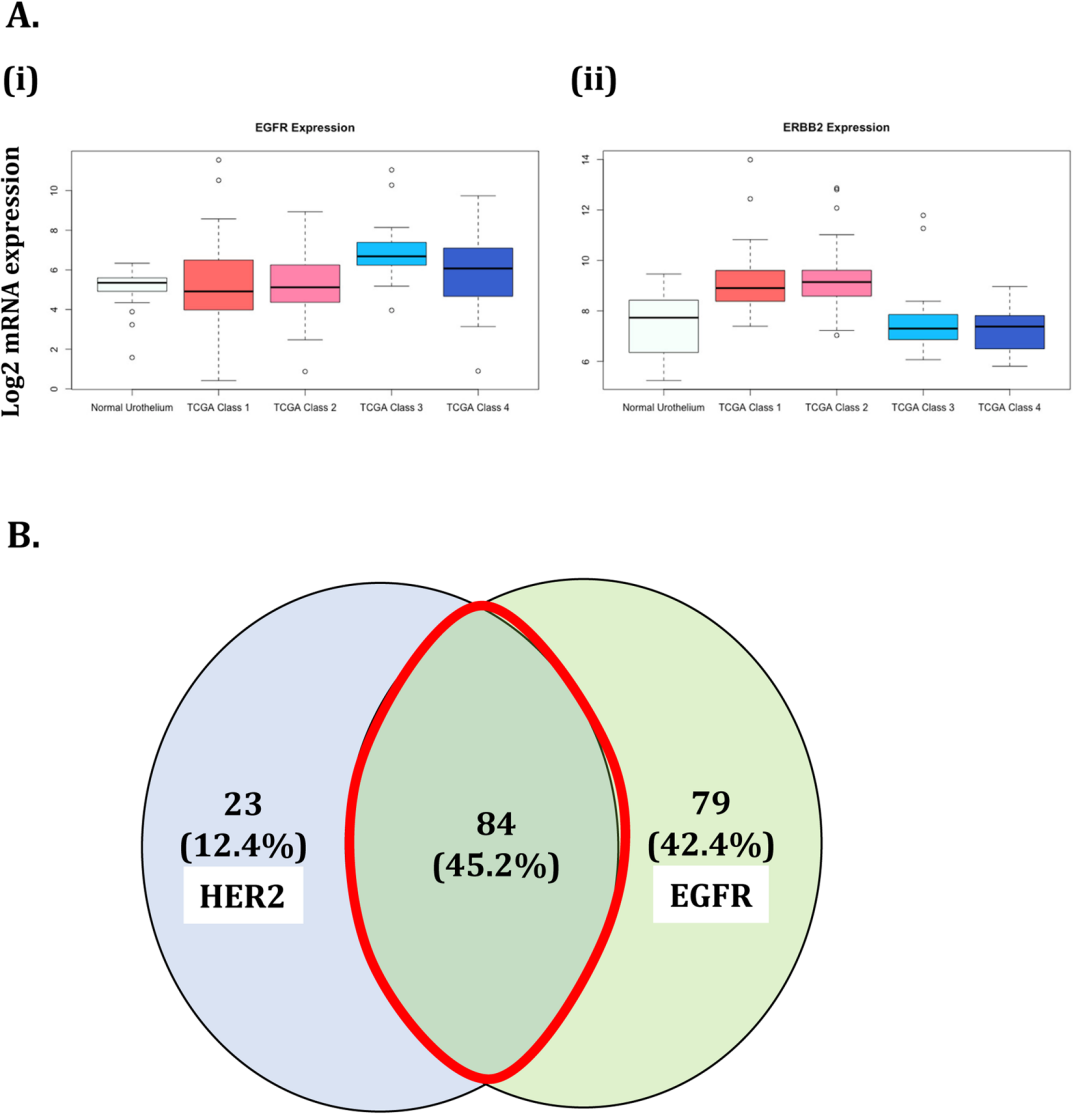
EGFR and HER2 expression in bladder cancer. (**A**) TCGA dataset was used to show increased expression of EGFR and HER2 in bladder cancer. (i) EGFR is significantly elevated in bladder cancer compared to normal urothelium (*P* < 0.01; ANOVA), specifically in ‘basal’ subtype classes 3 and 4. (ii) HER2 is significantly elevated in bladder cancer compared to the normal urothelium (*P* < 0.01; ANOVA), specifically in ‘luminal’ subtype classes 1 and 2. (**B**) Tissue microarrays BL2081 and BL806 (Biomax) were analyzed for the presence of EGFR and HER2 expression. 232 BC samples were stained, and 186 (80%) samples showed presence of either EGFR, HER2, or both. Of those, 79 (42.4%) samples and 23 (12.4%) showed only EGFR expression and only HER2 expression, respectively. 84 (45.2%) samples showed co-expression of EGFR and HER2.

The average gene expression in the TCGA provisional dataset confirmed that EGFR is elevated in bladder cancer and specifically in the ‘basal’ subtype classes 3 and 4 (p < 0.01, ANOVA). The dataset also confirmed that HER2 is elevated in bladder cancer compared to normal urothelium, more specifically in the ‘non-basal’ subtype classes 1 and 2 (p < 0.01, ANOVA) (Fig. 1A). EGFR and HER2 expression in human bladder cancer were further confirmed using commercially available tissue microarrays of human bladder cancer samples. A total of 232 bladder cancer samples were stained for EGFR and HER2, with 186 (80.1%) samples showing staining for either EGFR or HER2 or both. As seen in Fig. 1B, from the 186 samples stained, 23 (12.4%) samples stained only for HER2, 79 (42.4%) samples stained only for EGFR, and 84 (45.2%) samples stained for both EGFR and HER2, suggesting that co-targeting EGFR and HER2 for PIT is a viable option.

### EGFR and HER2 expression in bladder cancer cell lines

**Figure 2:**
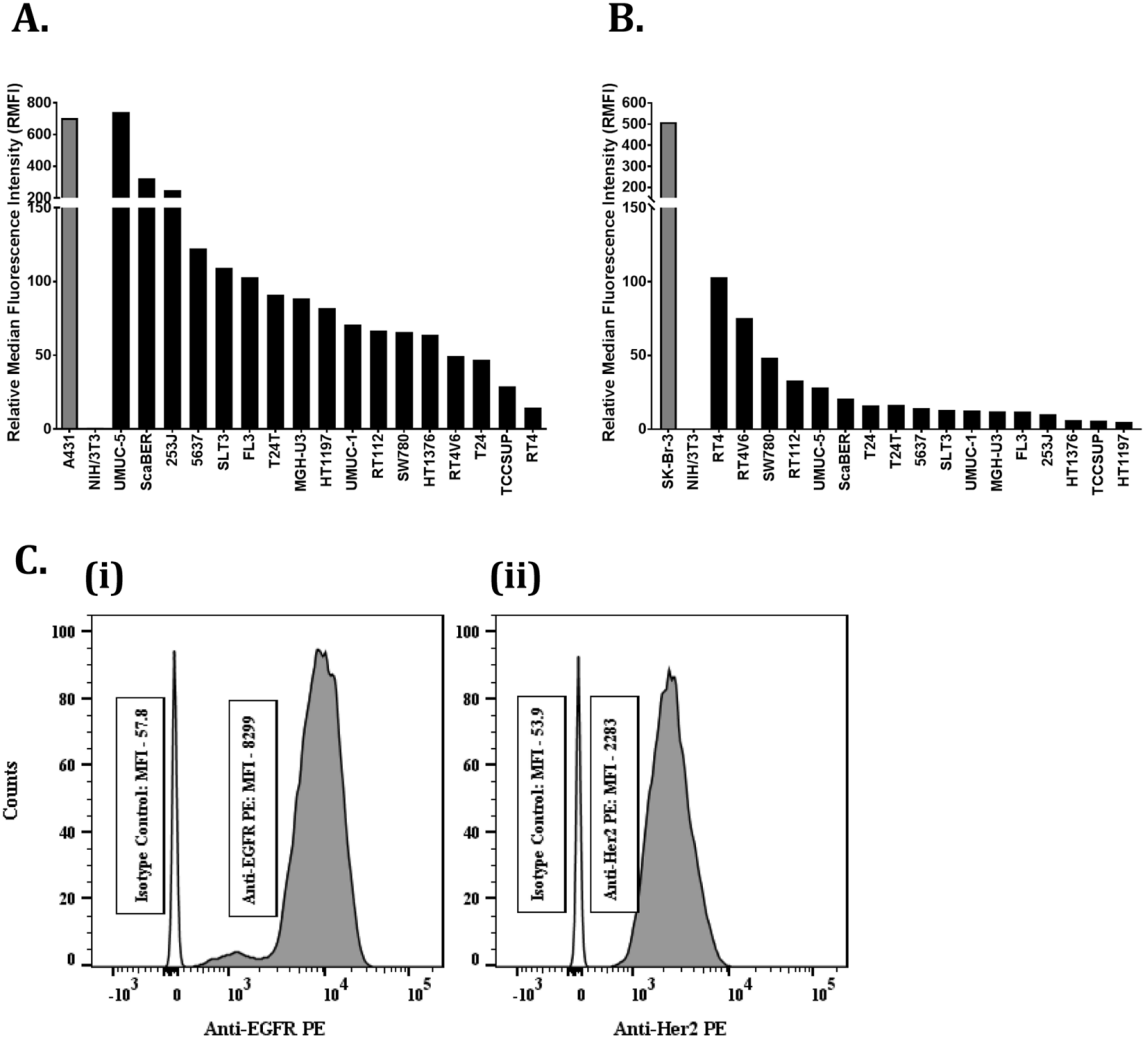
EGFR and HER2 expression in bladder cancer cell lines. Surface expressions of EGFR and HER2 on various bladder cancer cell lines were studied using flow cytometry. (**A**) Cell-surface expression of EGFR and (**B**) HER2 on the same set of bladder cancer cell lines. Briefly, single cell suspension of these cells was incubated with PE-tagged mouse MAb to HER2 or EGFR or PE-tagged mouse isotype control antibodies. The experiment was carried out at room temperature. At the end of 1 h incubation, the cells were washed and analyzed on the flow cytometer. The relative median fluorescence intensity (RMFI) for each cell line was calculated by the formula: $RMFI=\frac{Median\,Fluorescence\,Intensity\,(Anti-EGFR)\,or\,(Anti-Her2)}{Median\,Fluorescence\,Intensity\,(Corrosponding\,Isotype\,Control)}$ The RFMI of positive control is shown in grey, and the expression of both EGFR and HER2 in all conventional bladder cancer cell lines are shown in solid black bars. (**C**) Flow cytometry analysis showing (i) EGFR and (ii) HER2 expression in the SW780 cell line. Individual median fluorescence intensities (MFIs) of anti-EGFR PE, anti-Her2 PE and their corresponding isotype controls for SW780 cell line are indicated in the boxes.

The cell surface EGFR and HER2 expression on various bladder cancer cell lines was determined using flow cytometry. Railkar *et al*. from our laboratory has previously determined cell surface EGFR expression on a panel of human bladder cancer cell lines^6^ as shown in Fig. 2A. In this report, we additionally evaluated the same panel of cell lines for cell surface HER2 expression (Fig. 2B). The goal was to find a cell line with low-to-moderate cell surface EGFR and HER2 expression compared to the positive control and is tumorigenic in athymic nude mice.

Cell surface HER2 expression was determined by incubating cells with PE-conjugated anti-HER2 followed by MFI measurement using the flow cytometer. The breast cancer cell line, SK-Br-3, was used as a positive control, while the mouse fibroblast cell line, NIH/3T3, was used as a negative control for cell surface HER2 expression. As seen in the Fig. 2B, luminal cell lines such as RT4 and RT4V6 express relatively higher levels of cell surface HER2 compared to other cell lines tested here, albeit at a lower level compared to the breast cancer positive control cell line. Consistent with the literature, these cell lines showed very low levels of cell surface EGFR expression^15^. On the other hand, bladder squamous cell carcinoma cell lines such as UMUC-5 and ScaBER were highly enriched in cell surface EGFR, but showed very low levels of cell surface HER2. Looking across the panel of cell lines tested, we found that many expressed both EGFR and HER2 compared to the negative control and normal urothelial cells, but they were either enriched in EGFR or HER2. Only a few cell lines showed relative parity between the two cell surface proteins’ expression, which was more evident in a non-basal cell line such as SW780, which demonstrated low-to-moderate expression of both cell surface EGFR and HER2.

For SW780 cells, the MFI for EGFR and HER2 compared to their respective isotype controls were 143.5 (Fig. 2C(i)) and 42.35 (Fig. 2C(ii)), respectively, making the ratio of EGFR to HER2 expression about 3:1. This suggested that not only are these targets present in moderate numbers, but no one target is expressed in an “overwhelming” quantity, making it an ideal candidate for combination photoimmunotherapy. Given the low-to-moderate cell surface levels of both proteins and their ability to form tumors in athymic nude mice, SW780 cells were used for all our subsequent experiments.

### Panitumumab and trastuzumab specificity and co-localization on SW780 cells using immunocytochemistry

**Figure 3:**
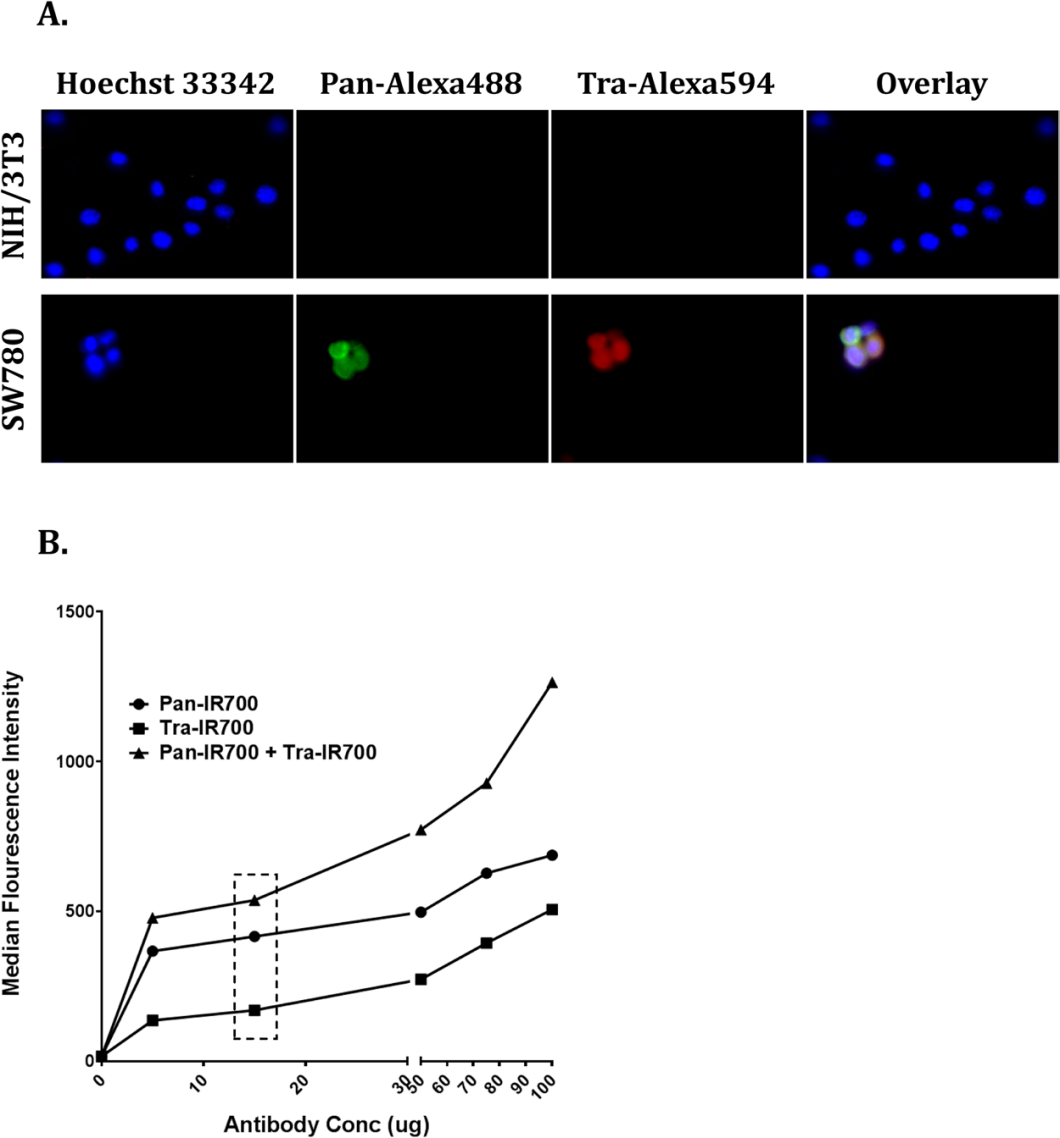
Panitumumab and trastuzumab are specific for human EGFR and HER2 and can bind their targets concurrently. (**A**) Simultaneous binding of panitumumab and trastuzumab to SW780 cells using immunocytochemistry. Panitumumab and trastuzumab were conjugated to Alexa488 and Alexa594, respectively. About, 3 × 10^4^ SW780 cells and NIH/3T3 cells were then incubated for 3 hours at room temperature with 50 μg/ml and 100 μg/ml of pan-Alexa488 and tra-Alexa594, respectively. NIH/3T3 cell line was used as a negative control. NIH/3T3 showed fluorescence in Hoechst 33342 (nuclear stain) but no fluorescence signals for Pan-Alexa488 and Tra-Alexa594, confirming the target specificity of the antibodies. SW780 cells showed fluorescence in both pan-Alexa488 and tra-Alexa594, and co-staining on same cells, confirming antibody specificity and their ability to bind targets concurrently. (**B**) Determining pan IR700 and tra IR700 concentrations to saturate binding sites. About, 1 × 10^6^ cells/100 µL were incubated for 1 hour at room temperature with either 0 µg, 5 µg, 15 µg, 50 µg, 75 µg, or 100 µg of pan IR700 alone, tra IR700 alone, or in combination (pan IR700 + tra IR700). The cells were washed and IR700 fluorescence was read using APC-Cy-7 filter of BD FACSCanto II flow cytometer machine. Flow cytometry shows that the antibody binding sites saturate about 15 µg/100 µL for both the antibodies. It also confirms concurrent binding of both antibodies to their targets on the same cell.

Panitumumab was conjugated to Alexa488, while Trastuzumab was conjugated to Alexa594 to not only confirm their specificity of binding to cell surface EGFR and HER2 on SW780, but also their ability to co-localize on the same cell. NIH/3T3 was used as a negative control. As shown in Fig. 3A, SW780 cells showed fluorescence signal in both pan-Alexa488 and tra-Alexa594 as well as co-localization of both signals on the same cells, confirming their target specificity and ability to concurrently bind their targets on the same cells. On the other hand, NIH/3T3 only showed fluorescence for nuclear stain, while no signal was detected in either pan-Alexa488 or tra-Alexa594, again confirming binding specificity of both the antibodies.

### Enhanced EGFR- and HER2-specific IR700 fluorescence signals with combined treatment of pan IR700 and tra IR700 *in vitro*

After cells were incubated at different concentrations of pan IR700 and tra IR700, IR700 fluorescence signals increased in a dose-dependent manner. The fluorescent signal began to saturate at about 15 μg of the respective single agent. When cells were treated with both pan IR700 and tra IR700, IR700 fluorescence signals again increased in a dose-dependent manner and exceeded the saturation level of single-agent treatment, even at lower concentrations of each antibody (Fig. 3B). Since, 15 μg of antibody showed binding saturation in both individual and combination treatments, it was subsequently used for all *in vitro* PIT experiments.

### *In Vitro* PIT: Combination therapy with pan IR700 and tra IR700 is superior to pan IR700 and tra IR700 treatment alone

**Figure 4:**
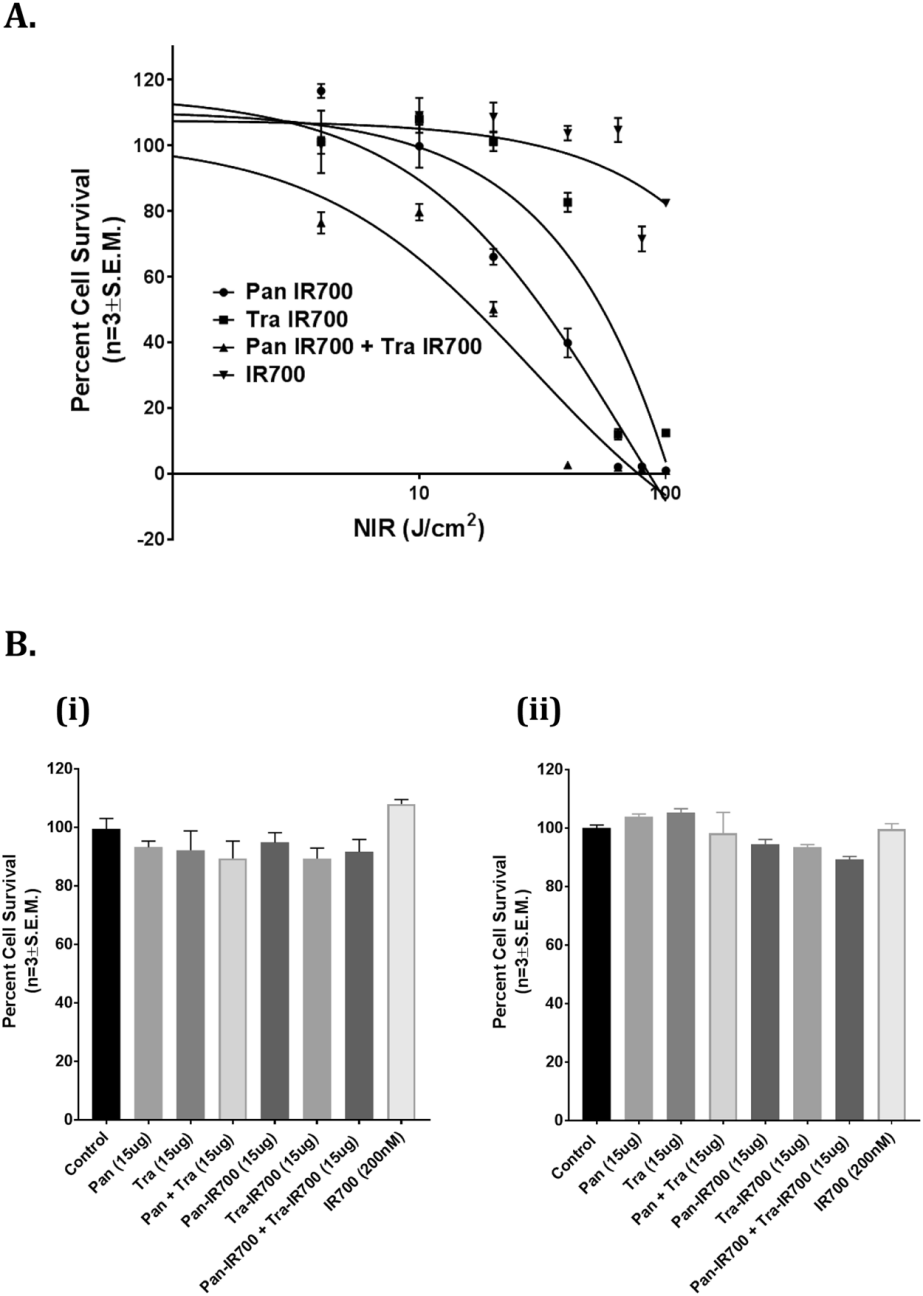
Combination of pan IR700 and tra IR700 mediated PIT is more cytotoxic than either agent alone *in vitro*. (**A**) Determination of LD_50_ of NIR light for combination PIT. 15 μg/mL (100 nM) of antibody was added to 1 × 10^5^cells/mL and incubated overnight. Cells were then irradiated with NIR light at 0 J/cm^2^, 4 J/cm^2^, 10 J/cm^2^, 20 J/cm^2^, 40 J/cm^2^, 64 J/cm^2^, 80 J/cm^2^, or 100 J/cm^2^ the following day. Cell survival was then measured 24 h later using Promega CellTiter-Glo Luminescent Cell Viability Assay. The LD_50_ for the combination PIT was determined to be 28.66 J/cm^2^ compared to 71.55 J/cm^2^ for pan IR700 with NIR arm, demonstrating potent phototoxicity in the combination group. LD_50_ for tra IR700 with NIR could not be determined due to insufficient cell death over 0–100 J/cm^2^ range of NIR irradiation. IR700 (200 nM) showed no overt toxicity. (**B**) Conjugated antibodies do not show any effects without NIR. IR700 conjugated or unconjugated panitumumab, trastuzumab, or panitumumab + trastuzumab are not cytotoxic at (i) 24 h or (ii) 72 h *in vitro*. 5 × 10^3^ SW780 cells/well were seeded overnight in a 96-well plate. Cells were then incubated with 100 nM antibody solution or 200 nM (100 nM pan or pan IR700 and 100 nM tra or tra IR700) in the combination well or 200 nM of IR700 Dye700Dx for 24 h or 72 h. Cell survival was measured using Promega CellTiter-Glo Luminescent Cell Viability Assay. No antibody or IR700 mediated cytotoxicity was observed between the groups at 24 h and 72 h.

SW780 cells were incubated overnight with 15 μg/ml (100 nM) of both pan IR700 and tra IR700, 15 μg/ml (100 nM) of pan IR700, 15 μg/ml (100 nM) of tra IR700, or 200 nM of IR700 alone. The cells were then irradiated with different amounts of NIR light, ranging from 0 J/cm^2^ to 100 J/cm^2^. The lethal dose-50 (LD_50_) for NIR light was then determined 24 hours later by measuring cell viability using CellTiter Glo reagent, which estimates cell survival by measuring the amount of ATP present. As shown in Fig. 4A, when cells were irradiated with NIR light after incubation with pan IR700 alone, the LD_50_ was found to be 71.55 J/cm^2^. However, when cells were irradiated with NIR light after incubation with both pan IR700 and tra IR700, the LD_50_ reduced significantly to 28.66 J/cm^2^, suggesting that the combination of both targets greatly enhances the effectiveness of photoimmunotherapy in causing cell death at lower amounts of NIR. The LD_50_ for the tra IR700 arm could not be determined, as there was not enough cell death even at 100 J/cm^2^ to accurately determine the LD_50_ value. There was no overt IR 700Dx toxicity observed in the presence of NIR light.

Additionally, antibody treatments by themselves showed no cytotoxicity when cells were treated for 24 hours (Fig. 4B(i)) and 72 hours (Fig. 4B(ii)) with 15 μg panitumumab, 15 μg trastuzumab, 15 μg of both panitumumab and trastuzumab, 15 μg pan IR700, 15 μg tra IR700, 15 μg of both pan IR700 and tra IR700, and 200 nM of IR700 without NIR exposure (*P* > 0.05 vs. control, Mann-Whitney *U* test).

### *In vivo* PIT

**Figure 5:**
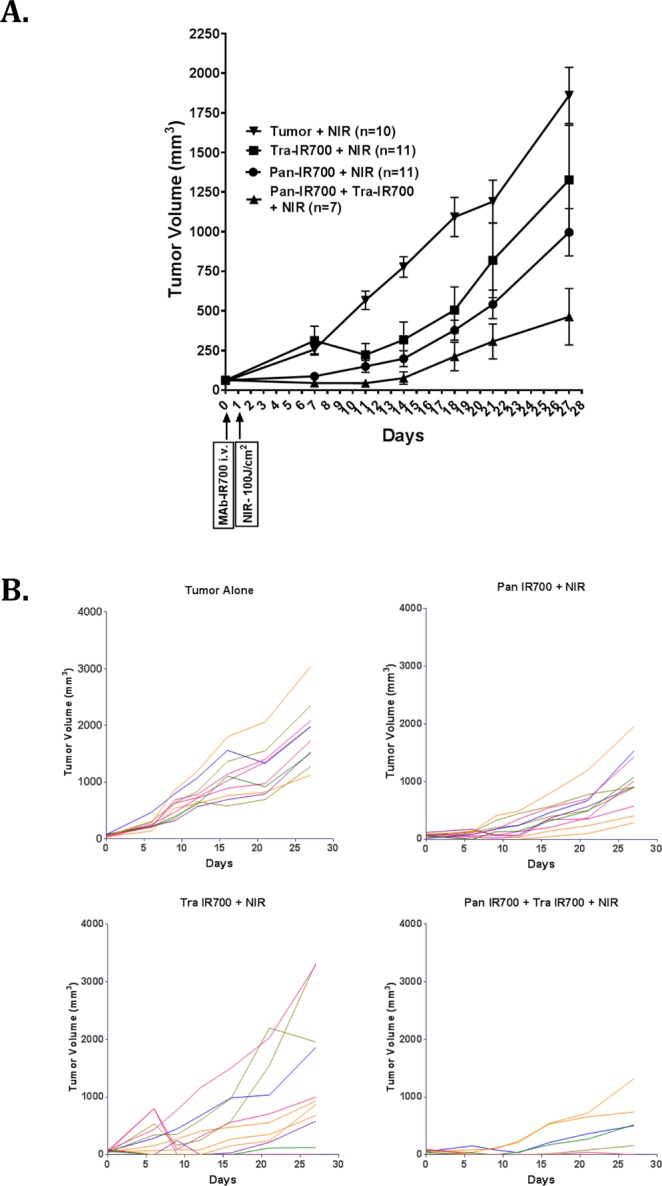
Effect of combination of pan IR700 and tra IR700 mediated PIT on SW780 xenografts. Combination treatment of pan IR700 and tra IR700 has stronger anti-tumor effects than single agents. Athymic Nu/Nu female mice of 5 weeks age were injected on the right thigh with 3 million/100 µl SW780 cells resuspended in 50% Matrigel in PBS. After 7 days, the tumors are about 50–100 mm^3^ in volume. The mice were randomized to groups of 10–11 animals/cohort and injected intravenously with 100 µg/100 µl of pan IR700, tra IR700 or pan IR700 + tra IR700 or 100 µl of vehicle (1x PBS). About 24 hours post injection, each mouse was restrained with 3% isoflurane and irradiated with (**A**) 100 J/cm^2^ of NIR light. Tumors were measured with external calipers twice a week for the next 28 days. Tumor volumes were measured by formula: (length) × (breadth)^2^ × 0.5. Data is represented as mean ± SEM (*P = 0.0335: pan IR700 + tra IR700 with NIR light vs. pan IR700 with NIR light; *P* = 0.068: pan IR700 + tra IR700 with NIR light vs. tra IR700 light; Mann-Whitney U test). (**B**) Tumor measurements of individual mice in each cohort treated with 100 J/cm^2^ of NIR light. There are about 10–11 mice in each cohort except in the combination PIT group of pan IR700 + tra IR700 where 4 out of 11 mice died following the NIR light treatment due to unrelated complications.

Tumor xenografts reached about 50–100 mm^3^ in volume approximately 7 days after subcutaneous injection of SW780 cells. To evaluate the efficacy of combination PIT, tumors were irradiated with 50 J/cm^2^ NIR light 24 h and 100 J/cm^2^ NIR light 24 h after the injection of antibodies in two separate experiments. Tumor volumes were then followed for 22–28 days. In the 50 J/cm^2^ NIR experiment, compared to the control group, pan IR700 with NIR light and pan IR700 + tra IR700 with NIR light showed significant tumor growth inhibition over 22 days. However, no clear difference was observed between the pan IR700 NIR group and the pan IR700 + tra IR700 groups, and so the experiment was repeated with 100 J/cm^2^ NIR (Supplemental Fig. 2). In the 100 J/cm^2^ NIR experiment, compared to the control group, pan IR700 with NIR light, tra IR700 with NIR light, and pan IR700 + tra IR700 with NIR light showed significant tumor growth inhibition over 28 days. However, the combination treatment involving pan IR700 + tra IR700 with NIR light showed better growth inhibition over time compared to the other treatment arms (n = 11 in pan IR700 with NIR light and tra IR700 with NIR light, n = 10 in Tumor with NIR light, and n = 7 in pan IR700 + tra IR700 with NIR light) (Fig. 5B). On Day 28, pan IR700 + tra IR700 with NIR light showed significant reduction in tumor growth compared to pan IR700 with NIR light (*P = *0*.*0335*; Mann-Whitney *U* test) (Fig. 5A). On the other hand, the pan IR700 + tra IR700 with NIR light demonstrated a strong trend towards greater tumor growth inhibition on Day 28 compared to the tra IR700 with NIR light group (*p* = *0*.*0687*; Mann-Whitney *U* test). While the combination PIT arm showed improved efficacy, it also had side effects with 4 mice dying within 48 h of the NIR light irradiation. This is likely due to profound cell lysis in these animals. The MAbIR700 treatment without NIR (pan IR700, traIR700 and pan IR700 + tra IR700) did not show any improvement in inhibition of tumor growth over control group (Supplemental Fig. 1).

### Possible acute tumor lysis induced by PIT

To evaluate potential treatment toxicity, a separate toxicity study was performed. In this study, 12 mice received combination PIT therapy, 2 were found dead within 16 h of NIR irradiation, and 3 were euthanized at 16 h of NIR irradiation secondary to appearing grossly edematous and severely lethargic. Edema was noticed at and around the NIR irradiation site, which was seen in all combination PIT treated mice, while no such changes were observed in PBS + NIR light treated mice. Pathologic review of the collected major organs in both combination and 1X PBS PIT treated mice revealed normal internal organs, while tumor necrosis was only seen in combination PIT arm. This study confirmed that combination PIT is not toxic to internal organs and only incurs damage to tumors (Supplemental Fig. 3).

**Figure 6:**
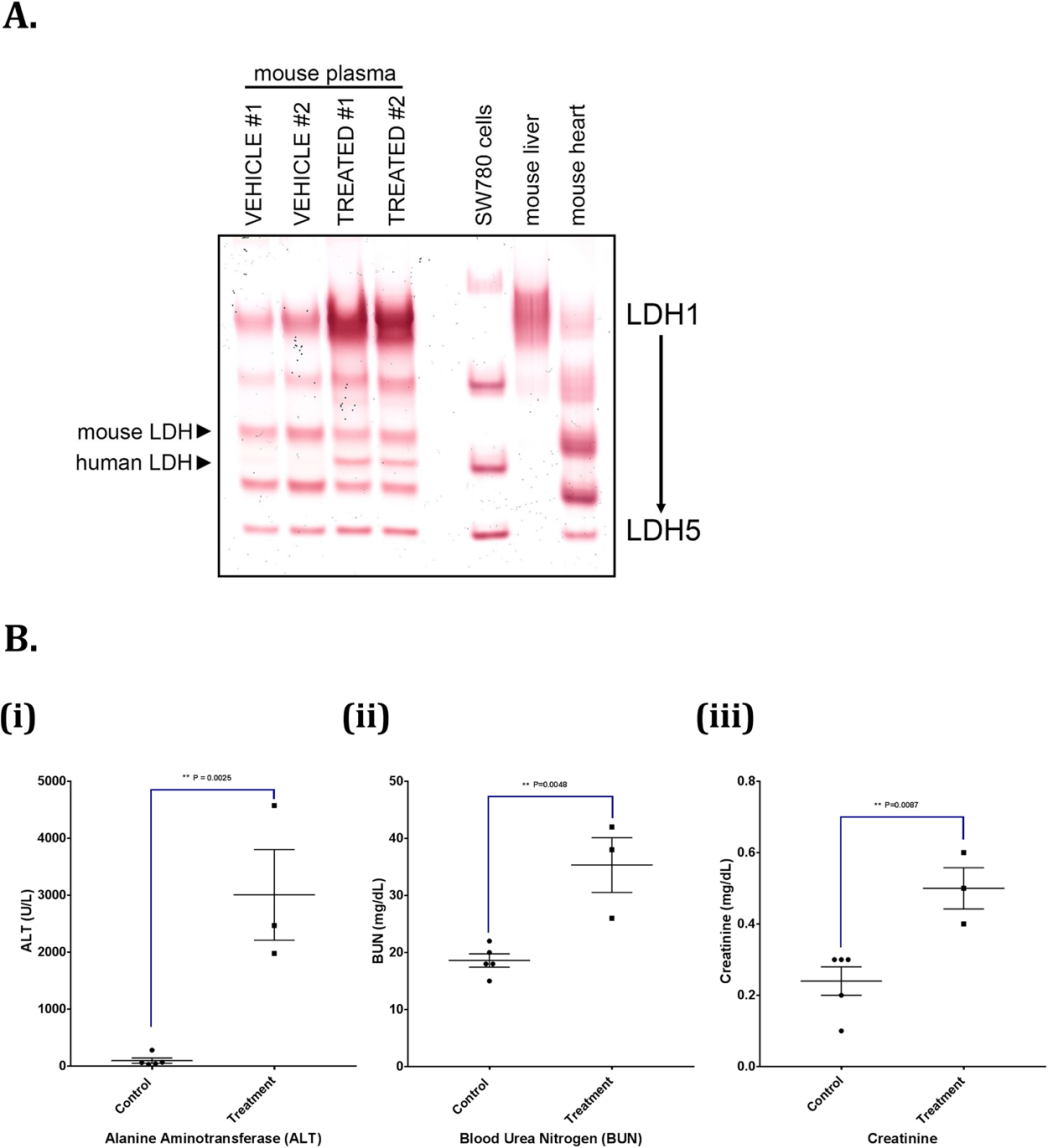
PIT induces tumor lysis. To study the possible toxicity of combination PIT, a total of 10 Athymic Nu/Nu female mice growing SW780 tumors on their right flank were injected intravenously with either 1XPBS or with 100 μg of pan IR700 + 100 μg of tra IR700. Twenty-four hours later, the mice were irradiated with 100 J/cm^2^ of NIR. The mice were euthanized 8 hours later, and blood was collected directly by puncturing the heart using 27 1/2 G needles into PST™ Microtainer^®^ tubes containing Lithium Heparin. Blood was mixed properly, centrifuged at 5600RPM for 15 minutes to separate plasma. This plasma was used for the following analyses. (**A**) Lactate dehydrogenase (LDH) measurement via in-gel activity assy. LDH of human origin is released in the plasma of the combination PIT treated mice. LDH in-gel activity assay was performed with plasma from 1X PBS and combination PIT treated mice. In the PIT-treated mouse plasma samples, human LDH-4 was observed as a band that was clearly resolved between the mouse LDH-3 and LDH-4 bands, demonstrating release of cellular LDH of human origin into the circulation following treatment indicating possible tumor lysis. Moreover, there was a significant increase in the mouse LDH-1 band in combination PIT treated mice indicating possible liver toxicity. (**B**) Blood chemistry is altered in the plasma of combination PIT mice. The blood chemistry was performed using VetScan^®^ Comprehensive Diagnostic Profile for the plasmas of vehicle and combination PIT treated mice. Increases in (ii) blood urea nitrogen (P = 0.0048) and (iii) creatinine (P = 0.0087) are indicative of massive tumor lysis syndrome after combination PIT treatment, whereas, an increase in (i) plasma alanine aminotransferase (P = 0.0025) corroborates the previous observation of possible liver toxicity.

We developed an in-gel LDH isozyme assay to evaluate the tissue and species specificity of LDH isozymes in plasma samples collected from 1X PBS or pan IR700 + tra IR700 based PIT treated SW780 xenograft-bearing mice. This assay allowed for resolution of mouse LDH-1 (consisting of four LDHB subunits) through LDH-5 (consisting of four LDHA subunits) (Fig. 6A; mouse liver and mouse heart samples), and four of the human LDH isozymes (human LDH-2–5) (Fig. 6A; SW780 cells). In the PIT-treated mouse plasma samples, human LDH-4 was observed as a band that was clearly resolved between the mouse LDH-3 and LDH-4 bands, demonstrating release of cellular LDH of human origin into the circulation following treatment. There was also a significant increase in mouse LDH-1 activity in the treated mouse plasmas, the pattern of which may be indicative of some liver damage in the treated mice however, no gross liver damage noted on pathology review as noted above.

The VetScan^®^ Comprehensive Diagnostic Profile of mice plasmas showed large increase in levels of Alanine Aminotransferase (ALT) (Fig. 6B(i)), blood urea nitrogen (BUN) (Fig. 6B(ii)) and creatinine (Fig. 6B(iii)), in PIT treated mice over 1X PBS treated mice. Other indications such as albumin, amylase and total protein showed slight changes in PIT treated mice (Supplemental Fig. 4) whereas no significant change was observed in the levels of alkaline phosphatase (AP), total bilirubin (TBIL), calcium, phosphate, glucose, sodium, potassium and globulin between the plasmas from the two groups of mice (Supplemental Fig. 5).

## Discussion

Here we present a novel approach to bladder cancer therapy using a combination of cell surface targets to enhance the anti-tumor effects of photoimmunotherapy. Taking advantage of the high molecular heterogeneity in bladder cancer, we targeted cancer that expresses both cell-surface EGFR and HER2. IR700-conjugated panitumumab and trastuzumab were used to target EGFR and HER2, respectively. We showed that the effects of combination PIT were far stronger than treatment with either agent alone in the presence of NIR irradiation, both *in vitro* and *in vivo*.

PIT is a targeted tumor therapy that involves IR700-conjugated MAb that is directed towards tumor cell surface receptors. The anti-tumor effects arise from NIR light-mediated activation of IR700 dye, a photo-absorber conjugated to MAbs. Although the mechanism underlying cytotoxicity from IR700 activation is not fully understood, it is believed to cause an acute weakening of the cell membrane leading to cytoplasmic swelling and cell death through necrosis^6^. Previously, we have shown that the basal/squamous subtype of bladder cancer, with enrichment in EGFR expression, can be very effectively targeted using EGFR-directed PIT^6^. However, we also noted that the EGFR-directed PIT is not very effective either *in* vitro or *in* vivo in BC with low cell surface expression of EGFR, as the efficacy of PIT is correlated with the number of IR700 dye molecules present on the cell surface^6^.

EGFR and HER2 are good candidate targets for PIT, as they both have been shown to be overexpressed in BC. Molecular subtyping has revealed that the basal/squamous subtypes are enriched in EGFR, while the luminal subtypes show enrichment in HER2^9,10^. However, this enrichment is not mutually exclusive and both EGFR and HER2 can be present on the same cell. This is evident from a study conducted by Chow *et al*. involving 245 human bladder cancer samples of various grades and stages that looked at EGFR and HER2 expression using immunohistochemistry. They showed that EGFR was present in 72.2% of tumors and HER2 was present in 44.5% of tumors. However, 33.9% of tumors co-expressed both EGFR and HER2, confirming that these two proteins can be present on the same tumor simultaneously^13^. Our TMA in human bladder cancer samples also confirmed this observation, where we found co-expression of both in 45.2% of the samples with varying levels of EGFR and HER2 staining. Interestingly, despite the presence of these two proteins on BC, BC does not show an “addiction” to them, as targeting of these receptors have failed to show any efficacy in phase II trials^16–18^. This might be because BC is likely not driven by these receptors and their respective downstream signaling pathways. Therefore, direct inhibition of these surface receptors is often clinically ineffective in tumor growth cessation.

The combination approach for PIT is greatly advantageous, as it allows for targeting a wide spectrum of bladder tumors more effectively, irrespective of enrichment of any one specific target. It has been shown previously that increasing the dose of MAb-IR700 correlates with increased killing at a constant NIR dose^7^. Combination PIT approach helps in homogeneous micro-distribution of MAb-IR700s throughout the tumor overcoming “binding site barrier”^19^. Ito and colleagues have also shown that the combination of two HER2 specific MAb-IR700 showed significantly stronger cell surface IR700 fluorescence than individual reagent alone^8^. This is consistent with our observation in Fig. 3B, where we show that treating tumor cells with the combination of pan IR700 and tra IR700 gives an amplified IR700 fluorescence compared to treatment with either agent alone. This greater IR700 fluorescence in the combination treatment group also bypasses the major limitation of ‘binding site’ saturation that occurs with a single antibody-IR700 conjugate. Due to the finite number of a given antigen on cell membranes, binding of MAb-IR700 is limited by that number. However, when binding of different MAb-IR700 conjugates to different antigens are utilized, the net number of cell surface-bound MAb-IR700 can be significantly increased^7,8^. As seen in Fig. 3, we demonstrate by flow cytometry and immunocytochemistry that pan IR700 and tra IR700 not only bind two different proteins on the same cell but also can bind their targets concurrently.

Previous studies have shown that increasing the number of bound MAb-IR700 conjugates on tumors leads to stronger phototherapeutic effects, and we demonstrate that a similar strategy can be used, both *in vitro* and *in vivo*, to effectively target BC as well^7,8,19^. In the *in vitro* setting (Fig. 4A), we show that when the BC cell line SW780 is targeted using both pan IR700 and tra IR700 with NIR light, the LD_50_ significantly reduced to 28.66 J/cm^2^ from 71.55 J/cm^2^ (the LD_50_ of the next most effective treatment arm of Pan IR700 with NIR light). Similarly, in the *in vivo* setting (Fig. 5A), we observed the strongest anti-tumor effects in the combination treatment arm involving both pan IR700 and tra IR700 with NIR light compared to the treatment arms with either agent alone. We did not see this initially with 50 J/cm^2^ NIR but we saw this effect when we increased the NIR to 100 J/cm^2^.

In our second *in vivo* experiment using 100 J/cm^2^, 4 of 11 (36%) mice in the tra IR700 with NIR treatment arm initially showed complete resolution of tumor, with tumor recurrence noted within 14 days of treatment. The initial robust response was likely secondary to the known anti-tumor effects of trastuzumab via NK cell-mediated antibody-dependent cell-mediated cytotoxicity (ADCC)^20,21^. Additionally, while the remaining mice in the tra IR700 with NIR arm demonstrated continued tumor growth, the mean tumor volume of the entire arm was likely “skewed” by the four outliers to make the p-value not significant when compared to the combination PIT arm. It should also be noted that the anti-tumor effects in both *in vitro* and *in vivo* settings were strictly mediated by IR700 activation by NIR light, as no cytotoxic or anti-tumor effects were observed when cells and mice were treated with MAb-IR700 without NIR, respectively (Fig. 4B and Supplemental Fig. 1).

In our experiments, combination PIT at 100 J/cm^2^ demonstrated toxicity with 4 of 11 mice dying within 48 h of 100 J/cm^2^ NIR light irradiation. These mice were noticed to have developed significant peri-tumoral edema and appeared lethargic, which was concerning for a strong, systemic inflammatory response from massive tumor necrosis and tumor lysis syndrome (TLS). A toxicity study confirmed peri-xenograft dermal edema with no organ damage to the major internal organs (heart, lungs, kidneys, spleen, liver, and bone marrow) supporting the hypothesis of development of a shock-like state from systemic inflammatory response to acute tumor necrosis (Supplemental Fig. 3). This is conceivable when considering the relatively large size of the xenograft in relation to the overall body size of a mouse^22^. Additionally, we did not dose escalate the two IR700-MAbs and it is possible that the combination of antibody conjugate doses at 100 J/cm^2^ were beyond the maximum tolerated doses for the animals. Although no toxicity was observed at 50 J/cm^2^, we also did not find the combination treatment efficacious at that light intensity. Careful dose escalation of MAb-IR700 concentrations and NIR dose may lead to finding the optimum dose for treatment effect with minimal toxicity. In humans, we strongly suspect that such systemic side effects will likely not occur for the following reasons: 1) the local bladder tumor will be infinitesimal relative to the entire body and will likely be mostly surgically resected prior to PIT treatment, and 2) unlike in mice experiments, there will be a Phase I dose escalation study to determine possible toxic doses of either IR700 labeled antibodies and/or dosage of NIR light. To date, in an ongoing trial of cetuximab IR700 NIR-PIT in recurrent head and neck cancers, no such toxic effects have been noted. (Clinicaltrials.gov identifier # NCT02422979).

Tumor lysis syndrome (TLS) is more frequently observed in liquid cancers such as Burkitt lymphoma, T-cell acute lymphoblastic leukemia, acute lymphocytic leukemia and high-grade non-Hodgkin’s lymphoma^23^. However, there have been cases of TLS in solid tumors as seen in metastatic colon carcinoma treated with cetuximab^24^. There are also reports of oncolytic virotherapy as well as the combination of chemotherapy with oncolytic clostridium bacteria inducing TLS in nude mice bearing solid tumor xenografts^25,26^.

Cells treated with PIT undergo rapid necrosis, and in a large solid tumor in mice, this rapid necrosis may lead to the onset of TLS. Hyperuricemia, hyperkalemia, hyperphosphatemia and hypocalcemia are some of the metabolic abnormalities resulting from TLS^27^. Unfortunately, we found it difficult to measure these electrolytes from the plasma of euthanized mice. For example, serum potassium levels of both untreated and PIT treated mice were higher than normal levels for rodents despite using cervical dislocation for euthanasia^28^. However, other metabolic effects of TLS such as increased plasma alanine transferase (ALT), blood urea nitrogen (BUN), creatinine, and lactate dehydrogenase (LDH), were observed 8 hours after the PIT treatment (Fig. 6A,B). Moreover, the presence of human LDH in the plasma of PIT-treated mice supports extrusion of tumor debris into the mouse circulation suggesting that TLS may be occurring secondary to PIT-induced rapid necrosis of tumor cells. In addition, the presence of mouse LDH isoforms specific to the liver in response to PIT may suggest acute hepatitis which is supported by the elevated levels of ALT, although no gross morphological changes were seen on liver necropsy. No incidence of TLS has been noted in humans in the setting of PIT, in the clinical trial mentioned above. Additionally, TLS is easier to manage in humans, requiring frequent intravenous hydration and allopurinol, interventions that are difficult to administer in murine xenografts^27^. Nevertheless, the results of our study suggest that further development of PIT in patients would require a dose escalation study for different combinations of MAb-IR700 conjugates and the NIR itself.

Bladder cancer is a multi-focal disease that requires treatment of the entire organ. PIT offers the ability to treat the bladder effectively via a transurethral approach. Although photodynamic therapy currently exists for human bladder cancer, it has been marred by complications, as it non-specifically targets normal urothelium^29–31^. Similarly, antibody conjugates such as the EGFR humanized chimeric monoclonal antibody C225 (cetuximab) conjugated to a benzoprophyrin derivative (verteprofrin) are also non-specific, making selective treatment of bladder cancer challenging^32^. Also, its efficacy has also been found to be limited^30^. Given the cell-selective nature of PIT, it can potentially be very effective against bladder cancer while sparing the normal urothelium.

In conclusion, the use of EGFR and HER2 as co-targets for PIT expands on our previous work of targeting EGFR-enriched bladder cancer. Since EGFR and HER2 are predominantly expressed in the basal and luminal subtypes of bladder cancer, respectively, combining both targets provide a novel and highly selective way of targeting bladder cancers that may have moderate expression of both. This then greatly broadens the scope of PIT in treating a much wider array of bladder cancers, irrespective of enrichment of any one target. Given the high molecular heterogeneity of bladder cancer, going forward, more cell surface targets need to be explored so that PIT can be personalized to every patient and target an even broader spectrum of bladder cancers.

## Supplementary information

Supplemental Figures
