# Supplementary material for: Targeting Epidermal Growth Factor Receptor (EGFR) and Human Epidermal Growth Factor Receptor 2 (HER2) Expressing Bladder Cancer Using Combination Photoimmunotherapy (PIT)
Source: Sci Rep. 2019 Feb 14;9:2084. doi: 10.1038/s41598-019-38575-x (PMC6375935; doi:10.1038/s41598-019-38575-x)
Supplement: Supplementary file 1 — Supplemental Figures [file 41598_2019_38575_MOESM1_ESM.pptx]

## Slide 1
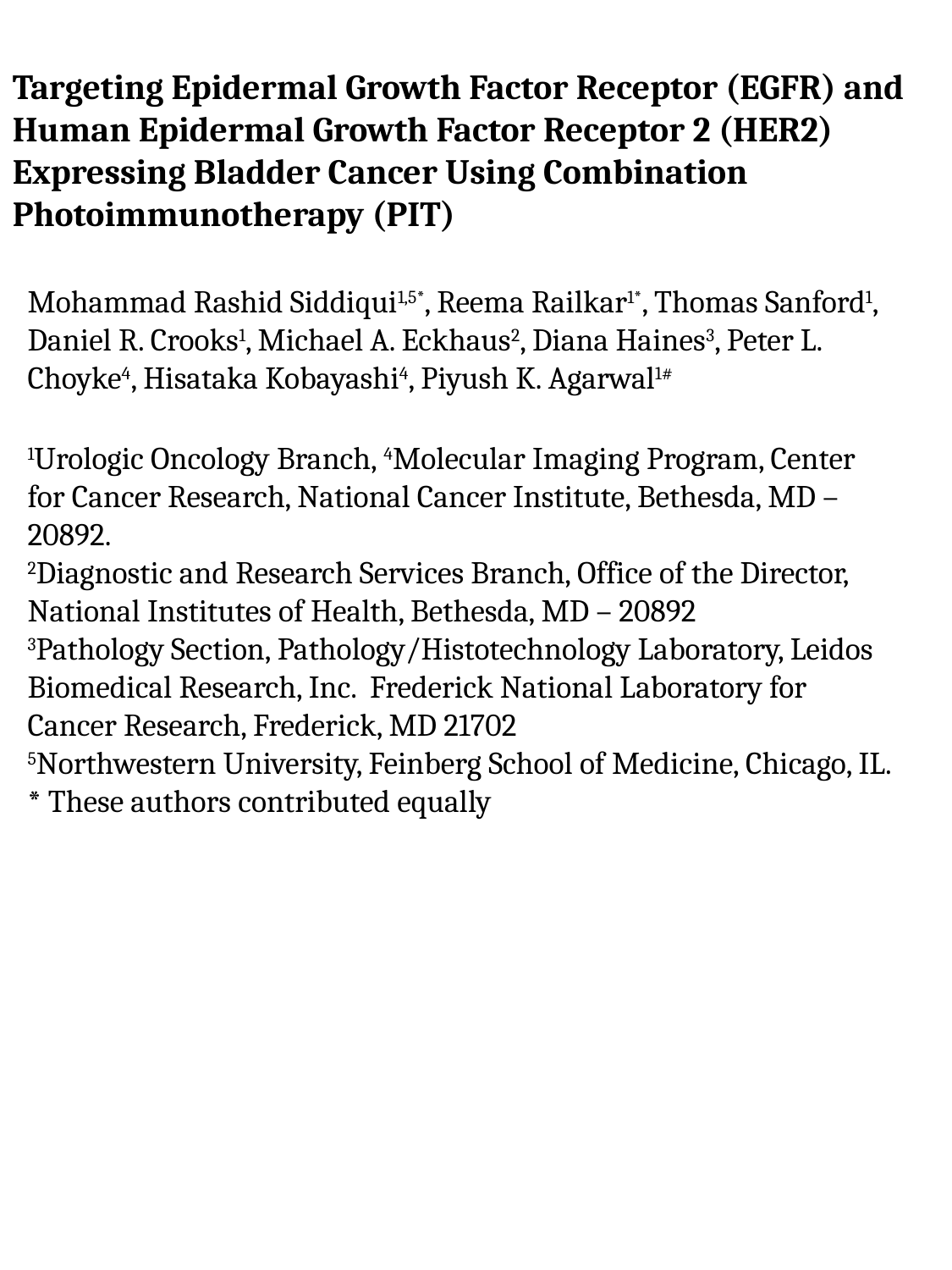

Targeting Epidermal Growth Factor Receptor (EGFR) and Human Epidermal Growth Factor Receptor 2 (HER2) Expressing Bladder Cancer Using Combination Photoimmunotherapy (PIT)
Mohammad Rashid Siddiqui1,5*, Reema Railkar1*, Thomas Sanford1, Daniel R. Crooks1, Michael A. Eckhaus2, Diana Haines3, Peter L. Choyke4, Hisataka Kobayashi4, Piyush K. Agarwal1#
1Urologic Oncology Branch, 4Molecular Imaging Program, Center for Cancer Research, National Cancer Institute, Bethesda, MD – 20892.
2Diagnostic and Research Services Branch, Office of the Director, National Institutes of Health, Bethesda, MD – 20892
3Pathology Section, Pathology/Histotechnology Laboratory, Leidos Biomedical Research, Inc. Frederick National Laboratory for Cancer Research, Frederick, MD 21702
5Northwestern University, Feinberg School of Medicine, Chicago, IL.
* These authors contributed equally

## Slide 2
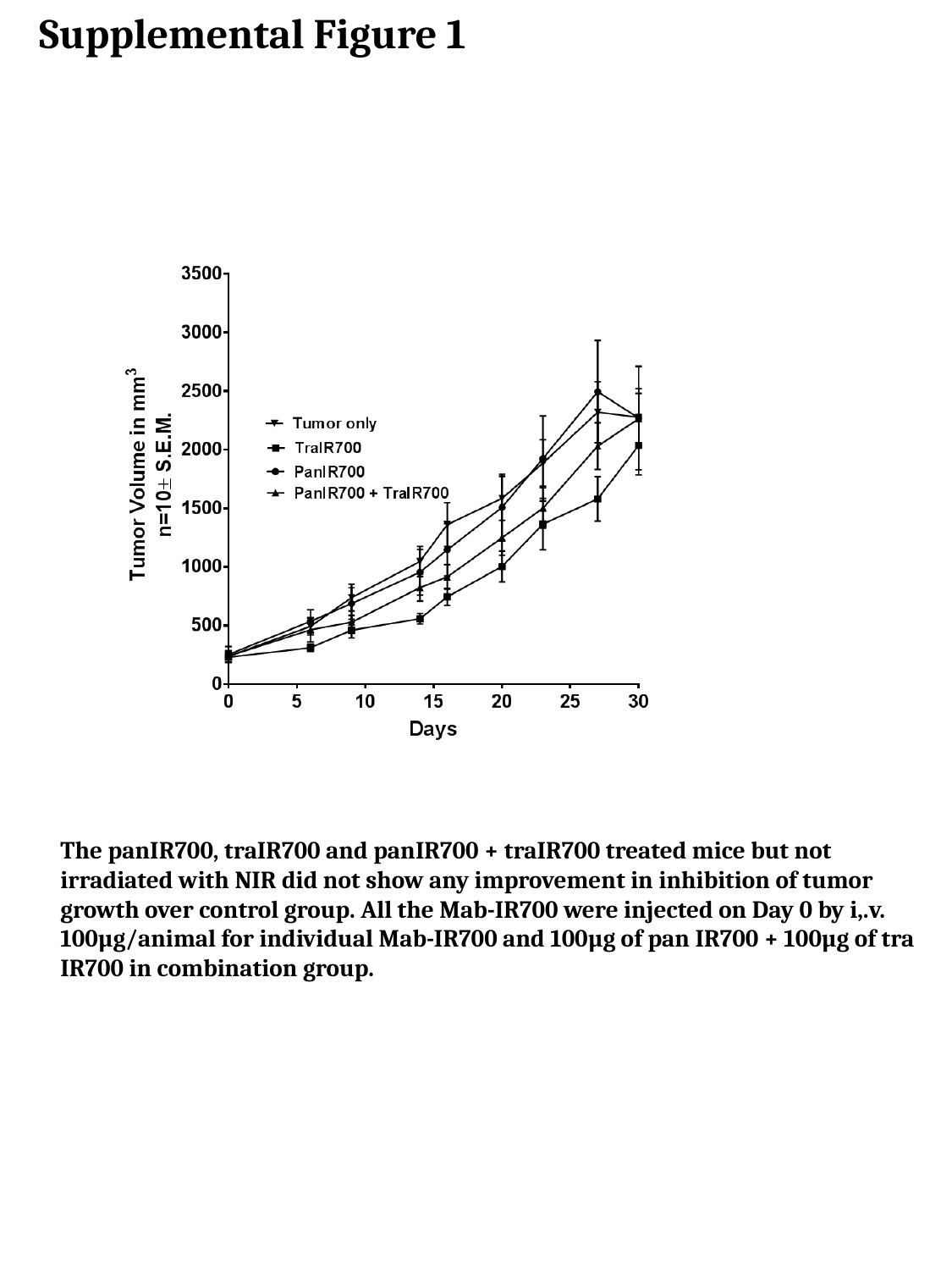

Supplemental Figure 1
The panIR700, traIR700 and panIR700 + traIR700 treated mice but not irradiated with NIR did not show any improvement in inhibition of tumor growth over control group. All the Mab-IR700 were injected on Day 0 by i,.v. 100µg/animal for individual Mab-IR700 and 100µg of pan IR700 + 100µg of tra IR700 in combination group.

## Slide 3
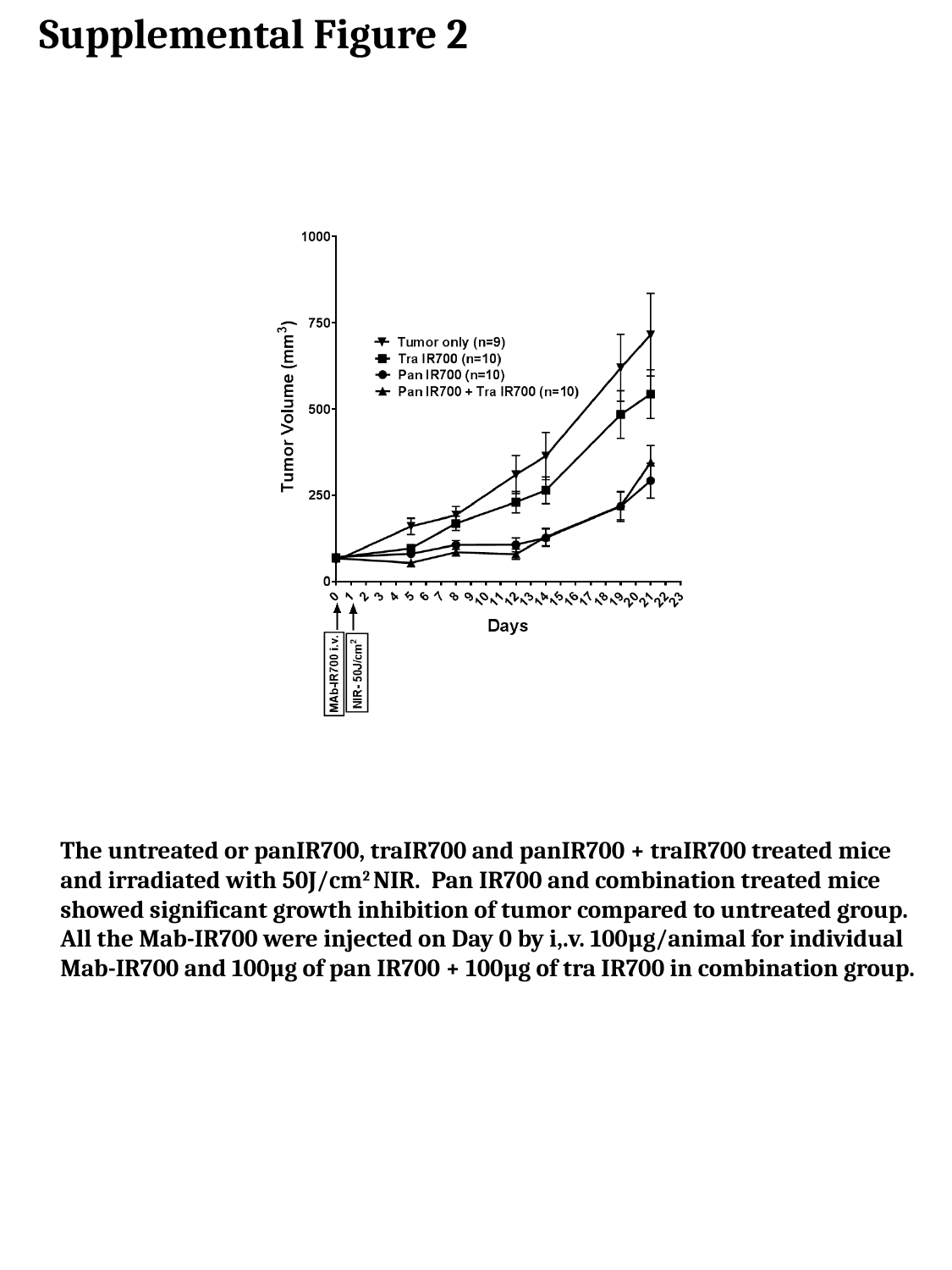

Supplemental Figure 2
The untreated or panIR700, traIR700 and panIR700 + traIR700 treated mice and irradiated with 50J/cm2 NIR. Pan IR700 and combination treated mice showed significant growth inhibition of tumor compared to untreated group. All the Mab-IR700 were injected on Day 0 by i,.v. 100µg/animal for individual Mab-IR700 and 100µg of pan IR700 + 100µg of tra IR700 in combination group.

## Slide 4
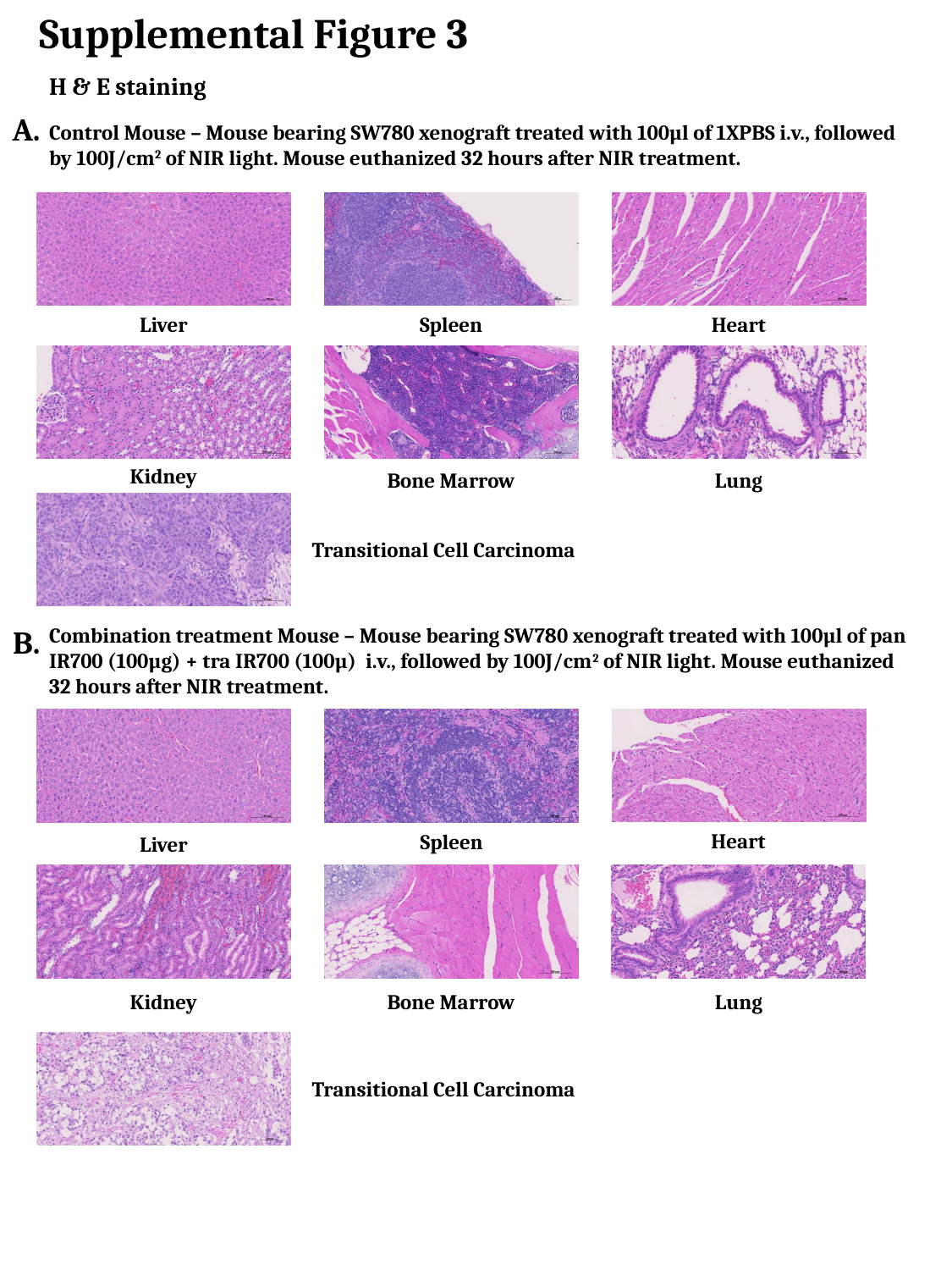

Supplemental Figure 3
H & E staining
A.
Control Mouse – Mouse bearing SW780 xenograft treated with 100µl of 1XPBS i.v., followed by 100J/cm2 of NIR light. Mouse euthanized 32 hours after NIR treatment.
Liver
Spleen
Heart
Kidney
Bone Marrow
Lung
Transitional Cell Carcinoma
B.
Combination treatment Mouse – Mouse bearing SW780 xenograft treated with 100µl of pan IR700 (100µg) + tra IR700 (100µ) i.v., followed by 100J/cm2 of NIR light. Mouse euthanized 32 hours after NIR treatment.
Heart
Spleen
Liver
Bone Marrow
Kidney
Lung
Transitional Cell Carcinoma

## Slide 5
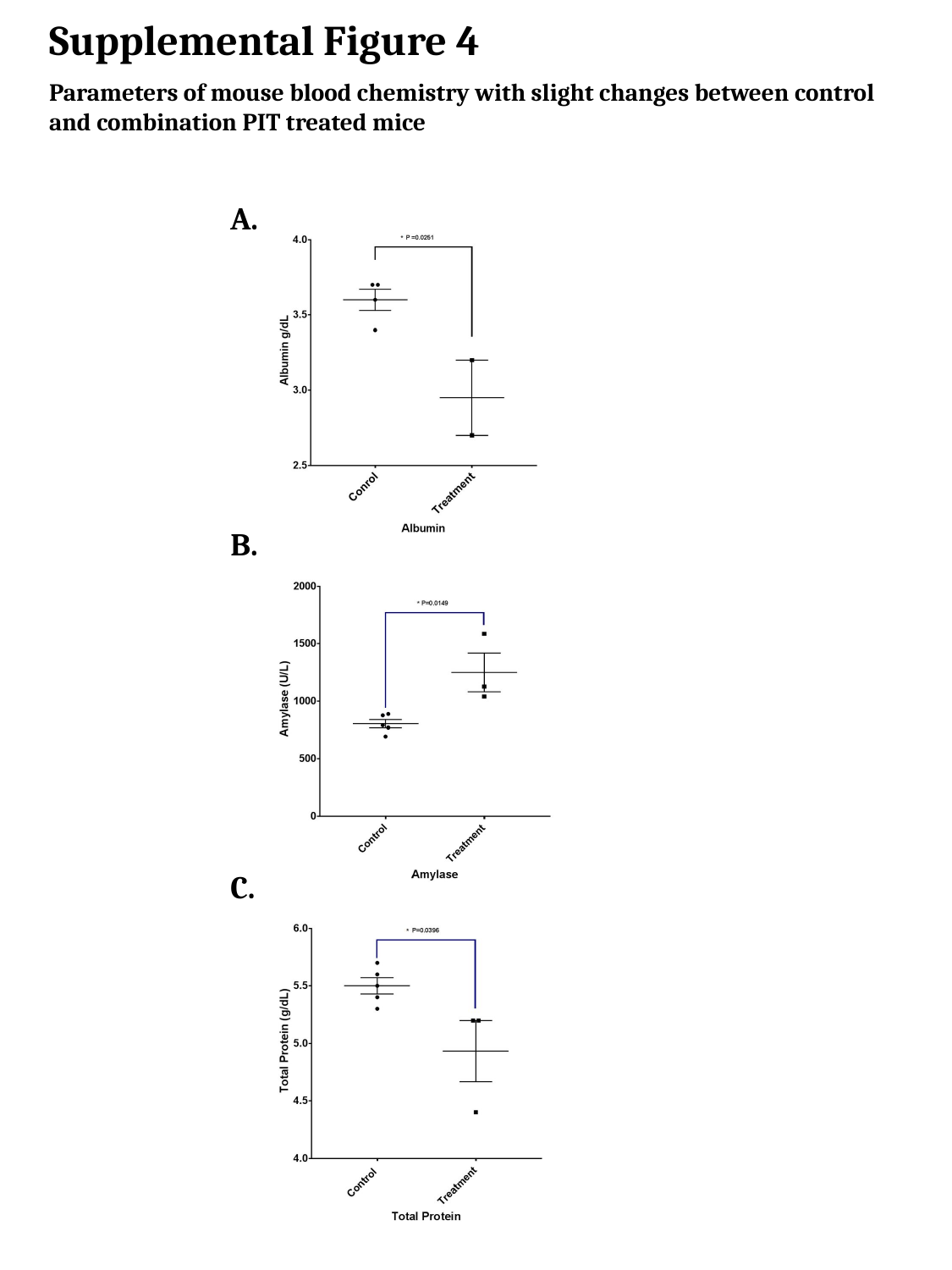

Supplemental Figure 4
Parameters of mouse blood chemistry with slight changes between control and combination PIT treated mice
A.
B.
C.

## Slide 6
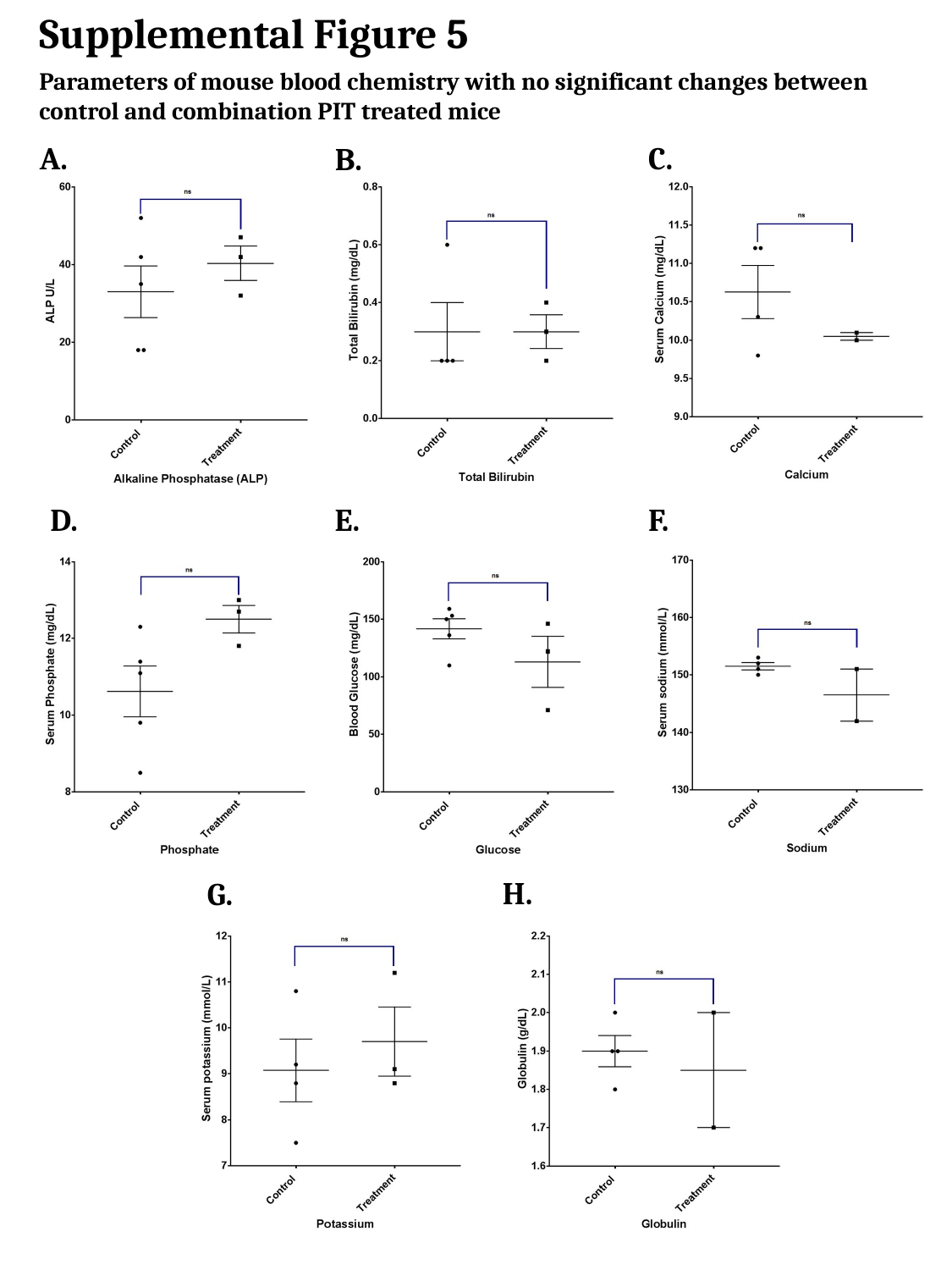

Supplemental Figure 5
Parameters of mouse blood chemistry with no significant changes between control and combination PIT treated mice
A.
C.
B.
D.
E.
F.
H.
G.
